# Characteristics of hardcore smokers in South Korea from 2007 to 2013

**DOI:** 10.1186/s12889-017-4452-z

**Published:** 2017-05-26

**Authors:** EunKyo Kang, Jung A. Lee, Hong-Jun Cho

**Affiliations:** Department of Family Medicine, Asan Medical Center, University of Ulsan College of Medicine, Seoul, Republic of Korea

**Keywords:** Smoking, Tobacco use, Korea

## Abstract

**Background:**

As the prevalence of smoking decreased in western countries, a significant proportion of smokers appeared to be particularly resistant to quitting- “hardcore” smokers. This study examines the characteristics of hardcore smokers in South Korea.

**Methods:**

We used the data from 2007 to 2013 from the Korea National Health and Nutrition Examination Survey. Hardcore smoking was defined as (1) smoking >15 cigarettes per day, (2) having no plans of quitting, and (3) having made no attempts to quit. Multiple logistic regression analyses were used to investigate the association between various sociodemographic variables and hardcore smoking.

**Results:**

The proportion of hardcore smokers among smokers did not change significantly from 23.1% in 2007 to 23.0% in 2013. None of the three characteristics of hardcore smokers for either gender showed a significant change from 2007 to 2013. Multiple logistic regression analysis showed that hardcore smokers were 1.64 times (95% confidence interval [CI], 1.28–2.11) greater among those aging 40–49 years than among those aging 19–29 years, and four times greater among men than women. Never-married smokers were less likely to be hardcore smokers than married ones (odds ratio 0.79; 95% CI, 0.66–0.96). Household income and education level did not have any significant association with the likelihood of a hardcore smoker.

**Conclusions:**

Hardcore smoking was more prevalent among men, unmarried men and those aging 40–49 years.

## Background

Tobacco smoking is a major risk factor for preventable premature death both worldwide and in South Korea (hereafter Korea) [1]. Smoking prevalence decreased from 35.1% in 1998 to 23.3% in 2014 among Korean adults (from 66.3% to 42.3% in men, and from 6.5% to 5.1% in women) [2]. Tobacco control policies, including tax increases, incremental expanding of smoke-free public indoor places, mass media campaigns highlighting the harmful effects of tobacco use, national quit lines, and national smoking cessation programs, contributed to this rapid drop in smoking prevalence [3].

Tobacco control policies have succeeded in decreasing smoking prevalence in most countries. However, regardless of a noticeable decrease in smoking prevalence over the past decades, the rate of decrease has attenuated in recent years [4]. To implement efficient smoking policies, we need to consider the concept of “hardcore smoking.” “Hardcore” smoker is a term that is used to generally refer to the proportion of smokers who are completely unwilling or unable to quit and are likely to remain so [5, 6].

There is no universally accepted definition for “hardcore smokers.” Moreover, the components that define a hardcore smoker are not all clearly associated with smoking cessation [7]. For instance, previous attempts to quit smoking were associated with the likelihood of trying to quit again but were not related to the probabilities of successfully quitting [8]. However, many characteristics of hardcore smokers have been identified, including a combination of nicotine addiction, intentions of quitting, expectations, and/or social disapproval, all of which have been found to predict smoking cessation [9, 10]. Therefore, it is necessary to discuss hardcore smoking to investigate the decline in quitting rates.

Most studies on hardcore smoking have been conducted in European countries, the USA, the EU [11], and New Zealand [12], where stages of tobacco epidemics are advanced [13]. However, only a few studies on hardening have been conducted in Asian countries where the tobacco epidemic was delayed, men’s smoking rates showed a flattening or downturn, and women’s smoking rates increased [14–16].

This study investigates the sociodemographic characteristics of hardcore smokers using nationally representative data.

## Methods

### Data source and study setting

This study used the data spanning 6 years, from 2007 to 2013, from the Korea National Health and Nutrition Examination Survey (KNHANES), a nationwide cross-sectional survey conducted every year with a target population comprising nationally representative non-institutionalized adults (aged ≥19 years) in Korea [17]. The survey developed a continuous program with approximately 10,000 individuals each year except for 2007, where the total number of participants was half of that in other years because of the survey being conducted mid-year (from July through December).

Respondents were selected using a multi-stage clustered probability sampling system. In the survey, primary sampling units (PSUs) were drawn from geologically defined PSUs for the entire nation. A PSU consisted of an average of 60 households, and 20 final target households were sampled for each PSU using systematic sampling. In the selected households, individuals aged *≥*1 year were targeted. The sample weights were constructed for sample participants to represent the Korean population by accounting for the complex survey design, non-respondents, and post-stratification [17].

On average, the total number of participants selected each year was 73,414, whereas the response rates from 2007 to 2013 ranged from 74.3% to 81.9% (average response rate: 78.5%). This study was conducted using data from 58,423 respondents.

Health component interviews (housing characteristics, medical conditions, socioeconomic status, quality of life, etc.) were performed face-to-face by a trained medical interviewer. Smoking, alcohol use, and related health behavior components were based on the information provided by a self-report questionnaire.

### Ethics statement

The anonymized data were obtained from KNHANES (https://knhanes.cdc.go.kr/knhanes/eng/index.do/). No specific ethical approval was required for their use.

### Variables

#### Smoking status

Current smokers were defined as respondents who consumed ≥100 cigarettes in their lifetime, and smoked cigarettes “daily” or “sometimes.” Ex-smokers were those who consumed ≥100 cigarettes in their lifetime but do not smoke “now.” Never-smokers were those who did not consume cigarettes in their lifetime. Smoking period was not investigated during 2007–2009 study period.

#### Hardcore smoking status

We selected three characteristics of a hardcore smoker that were related to continued smoking [18]: (1) High daily cigarette consumption, defined as smoking ≥15 cigarettes per day; (2) no intention of quitting, defined as never planning to quit; and (3) having made no attempts to quit smoking in the past year that lasted longer than 24 h. These three characteristics were known to predict future quitting behavior [18]. “Hardcore” smokers were defined as smokers who possessed all three characteristics described above.

#### Sociodemographic characteristics

The sociodemographic variables included age, gender, household income, educational attainment, and marital status. Age was classified into five groups: 19–29, 30–39, 40–49, 50–59, and >59 years old. Economic status was classified on the basis of the top two quartiles and bottom two quartiles of household incomes. Educational attainment was divided into the following four groups: middle school or lower, high school, college or university, and graduate school or higher. Marital status was defined as married/cohabiting, divorced/widowed/separated, or never-married.

### Statistical analysis

We analyzed the data in two ways. First, we examined the 7-year trend of the age-adjusted proportion of hardcore smokers using linear-by-linear association analysis. Second, we used multiple logistic regression analysis to estimate the associations between hardcore smoking, its characteristics, survey years, and sociodemographic variables. In the multiple logistic regression analysis, we entered all independent variables into the model simultaneously. We evaluated the variance inflation factor and found no multicollinearity among independent variables. We performed separate analyses for both genders using SPSS version 21.0 given the large gender difference in smoking prevalence.

## Results

Sociodemographic characteristics of the study subjects are shown in Table 1. Although smoking prevalence decreased from 25.0% in 2007 to 23.2% in 2013, this change was not significant (*p* = 0.208). However, smoking prevalence basically increased for females between 2007 and 2013 (5.3% and 5.7% respectively; *p* = 0.048). Smoking prevalence was greater in the 30–39 years age group (33.3%), low-moderate income group (27.9%), and never-married group (33.2%). Table 2 shows the proportion of hardcore smokers and their characteristics by gender. The proportion of hardcore smokers was 23.1% in 2007 and showed an insignificant change to 23.0% in 2013. The proportion of male hardcore smokers was more than two times greater than that of female hardcore smokers, and the trend did not change over the years. The difference in hardcore smokers between genders was mainly driven by the difference in heavy smokers.

**Table 1:** Weighted participant characteristics according to smoking status (*N* = 40,456)

	Total N (%)	Smoking rate, %	Male smoking rate, %	Female smoking rate, %
Number of smokers/Total participants		8415/40,456	7141/17,137	1274/23,319
Year				
2007	2980 (7.6)	25.0	45.1	5.3
2008	6798 (15.4)	27.3	47.7	7.3
2009	7470 (15.5)	26.6	46.7	6.9
2010	6256 (15.5)	26.9	48.1	6.1
2011	6023 (15.6)	26.3	46.8	6.5
2012	5591 (15.2)	25.0	43.3	7.4
2013	5338 (15.2)	23.2	41.4	5.7
Age (years)				
19–29	5065 (19.8)	29.0	46.2	10.5
30–39	7720 (21.1)	33.3	57.8	7.6
40–49	7602 (21.8)	27.6	49.3	5.5
50–59	7320 (17.6)	23.3	41.9	4.9
≥ 60	12,749 (19.6)	14.8	28.5	4.5
Sex				
Male	17,137 (49.3)	45.7		
Female	23,319 (50.7)	6.5		
Household income				
Low	8108 (16.1)	23.7	44.8	7.6
Low-moderate	10,160 (26.2)	27.9	48.3	8.6
Moderate-high	10,650 (28.8)	27.2	47.5	5.8
High	10,886 (28.8)	23.8	41.8	4.6
Education				
Middle school or less	10,863 (19.2)	16.4	39.3	5.7
High school	4408 (10.0)	26.6	45.0	8.3
College or university	13,716 (39.4)	30.0	49.4	8.4
Graduate school or more	11,385 (31.4)	26.0	43.6	4.2
Marital status				
Married/ cohabited	29,204 (67.9)	24.5	44.0	4.7
Divorced/separated/ bereaved	5517 (10.7)	19.3	54.1	9.7
Never married	5611 (21.4)	33.2	48.5	10.6

**Table 2:** Weighted proportion of hardcore smokers with hardcore smoking measures among smokers (*N* = 8415)

	Hardcore smoking measures	2007	2008	2009	2010	2011	2012	2013
Weighted, Age-Adjusted	Hardcore smoker*	23.1	23.7	23.4	26.6	25.8	25.6	23.0
	Heavy smoker	57.3	54.4	55.1	56.8	59.0	53.1	57.0
	No plan to quit in 6 months	63.3	60.5	61.3	54.0	56.9	56.4	56.6
	No quit attempt in past 12 months	39.2	43.7	42.8	45.6	44.6	44.0	42.9
Male, Weighted, Age-unadjusted	Hardcore smoker**	25.1	25.4	25.5	29.0	27.7	28.8	25.3
	Heavy smoker	61.4	59.0	59.6	62.1	63.4	59.6	62.1
	No plan to quit in 6 months	62.3	60.0	60.7	52.4	57.3	55.2	55.9
	No quit attempt in past 12 months	40.1	42.6	42.1	46.6	43.1	45.5	43.0
Female, Weighted, Age-unadjusted	Hardcore smoker**	6.9	11.8	9.3	8.0	12.5	7.3	6.2
	Heavy smoker	19.9	22.8	24.6	15.2	24.7	10.9	16.4
	No plan to quit in 6 months	70.9	61.6	64.0	64.4	50.5	62.1	58.1
	No quit attempt in past 12 months	32.1	43.5	42.2	36.7	49.5	39.9	39.3

*P for trend for hard core smokers = 0.573

**P for trend for male hardcore smokers = 0.697; p for trend for female hardcore smokers = 0.311

Table 3 shows the results of multiple logistic regression analysis, which identifies how particular variables contribute to the likelihood of being a hardcore smoker. No significant difference in the odds ratio (OR) of hardcore smokers was found between 2013 and 2007 (OR = 0.91; 95% CI [0.69, 2.10]). Hardcore smokers were nearly four times greater among men than among women (OR = 3.90; CI [3.07, 4.97]). Compared with smokers aged 19–29 years, those aged 40–49 years (OR = 1.64; CI [1.28, 2.11] and 50–59 years (OR = 1.51; CI [1.15, 1.98]) were significantly more likely to be hardcore smokers. Never-married individuals were less likely to be hardcore smokers than married ones (OR = 0.79; 95% CI [0.66–0.96]). Also, after considering the interaction between age and marital status, the never-married and divorced/separated/bereaved individuals were less likely to be hardcore smokers than married/cohabited individuals (OR = 0.76; 95% CI [0.61–0.95] in never-married group, OR = 0.80; 95% CI [0.68–0.93] in divorced/separated/bereaved group). Household income and education level did not have any significant association with one’s likelihood of becoming a hardcore smoker.

**Table 3:** Multivariate logistic regression analysis for hardcore smokers and hardcore smoking measures according to socioeconomic status (all odds ratios are adjusted for other variables in the models)

	Hardcore smoker	Heavy smoker	No plan to quit in 6 months	No quit attempt in past 12 months
	OR (95% CI)	OR (95% CI)	OR (95% CI)	OR (95% CI)
Year				
2007	1	1	1	1
2008	1.06 (0.81–1.39)	0.88 (0.68–1.12)	1.15 (0.90–1.46)	1.22 (0.99–1.52)
2009	1.06 (0.81–1.39)	0.90 (0.70–1.15)	1.13 (0.88–1.44)	1.15 (0.93–1.41)
2010	1.15 (0.87–1.51)	0.84 (0.66–1.07)	1.29 (1.00–1.67)	1.07 (0.86–1.33)
2011	1.06 (0.79–1.41)	0.92 (0.71–1.18)	1.22 (0.93–1.59)	0.93 (0.74–1.15)
2012	1.14 (0.86–1.52)	0.74 (0.57–0.97)	1.27 (0.98–1.64)	1.08 (0.85–1.37)
2013	0.91 (0.69–1.20)	0.82 (0.63–1.05)	1.14 (0.88–1.48)	1.01 (0.81–1.27)
Age (years)				
19–29	1	1	1	1
30–39	1.27 (1.01–1.60)	1.59 (1.31–1.94)	1.00 (0.83–1.20)	1.27 (1.05–1.54)
40–49	1.64 (1.28–2.11)	2.31 (1.86–2.87)	1.23 (1.00–1.50)	1.51 (1.24–1.85)
50–59	1.51 (1.15–1.98)	2.47 (1.95–3.14)	1.11 (0.89–1.38)	1.43 (1.14–1.79)
≥ 60	1.10 (0.82–1.48)	0.96 (0.75–1.23)	1.13 (0.90–1.44)	1.33 (1.04–1.71)
Household income				
Low	1	1	1	1
Low-intermediate	0.98 (0.83–1.15)	0.89 (0.77–1.02)	0.98 (0.85–1.13)	0.95 (0.82–1.10)
High-intermediate	0.88 (0.73–1.05)	0.90 (0.77–1.05)	0.97 (0.83–1.13)	1.00 (0.86–1.17)
High	0.88 (0.71–1.09)	0.70 (0.58–0.85)	0.98 (0.82–1.17)	0.94 (0.77–1.13)
Sex				
Female	1	1	1	1
Male	3.90 (3.07–4.97)	7.26 (6.00–8.79)	1.14 (0.98–1.33)	1.22 (1.05–1.42)
Education				
Middle school or less	1	1	1	1
High school	1.15 (0.91–1.45)	1.64 (1.30–2.08)	0.85 (0.70–1.04)	0.94 (0.75–1.19)
College or University	1.03 (0.83–1.26)	1.35 (1.09–1.65)	0.87 (0.73–1.03)	0.74 (0.60–0.91)
Graduate school or more	0.83 (0.65–1.05)	0.96 (0.77–1.20)	0.79 (0.65–0.97)	0.67 (0.53–0.84)
Marital status				
Married/cohabited	1	1	1	1
Widow/divorced/separated	1.09 (0.85–1.39)	1.09 (0.89–1.32)	1.21 (1.00–1.47)	1.14 (0.92–1.40)
Never-married	0.79 (0.66–0.96)	0.62 (0.53–0.73)	0.98 (0.84–1.15)	0.94 (0.81–1.10)
Marital status*				
Married/cohabited	1	1	1	1
Widow/divorced/separated	0.80 (0.68–0.93)	0.65 (0.58–0.74)	1.11 (0.98–1.27)	1.12 (0.97–1.29)
Never-married	0.76 (0.61–0.95)	0.57 (0.48–0.68)	1.01 (0.84–1.22)	0.95 (0.80–1.14)

* Further adjusted for interaction between age and marital status.

## Discussion

This study showed that neither the proportion of hardcore smokers nor the general smoking prevalence changed significantly for either gender from 2007 to 2013. In addition, there were no changes in the three characteristics of hardcore smokers: proportion of heavy smokers (≥15 cigarette/day), number of smokers who had attempted to quit in the past 12 months, and the number of smokers with no plans of quitting in 6 months. These findings are consistent with previous studies sharing a similar definition of hardcore smokers [5, 19].

The proportion of hardcore smokers among smokers was 23.0% in 2013—which was similar to that in an Italian study (23.5%) conducted in 2007 but much greater than that in a Californian study (5.2%) conducted in 2000 [20] using the same definition of hardcore smokers. The prevalence of hardcore smokers was different according to the definition and stage of the tobacco epidemic. Males were four times more likely to be hardcore smokers than were females. Although this was reported in most studies [20–23], the difference was much greater in Korea. This was mainly because of the difference among heavy smokers. Korean women had delayed initiation of cigarette smoking and smoked fewer cigarettes than their male counterparts [2]. In addition, gender differences are also generally driven by the lower social acceptability of female smokers than male smokers, which could have been the case in Korea [16, 24]. Hardcore smokers were more prevalent in the middle-aged groups (30–59 years). This finding is different from those of many western studies where hardcore smokers are more prevalent among the older age groups [23, 25] but is consistent with the study conducted in Italy [26]. This might be because of the delayed transition of the smoking epidemic in South Korea and Italy [13]. The never-married were less likely to be hardcore smokers than were the married (OR 0.79; 95% CI, 0.66–0.96). However, in the case of marital status, age, and other variables, interactions can be seen with age or other variables. After considering the interaction between age and marital status, we found that it was still significant. Comparing the married and cohabiting group with the divorced/separated/bereaved groups showed that the latter had lower hardcore smoking rates than the former. Never-married smokers were less likely to be hardcore smokers than married or cohabiting smokers, which is consistent with a Canadian study [27] but different from the study conducted in Hong Kong [15]. Our findings showed that economic status and education level were not strongly related to being a hardcore smoker. Although this result was comparable with that of some studies [14, 28], no significant association was found in this study and others [5, 19, 21, 23, 27]. This finding is interesting because many prior studies showed socioeconomic status disparities in hardcore smoking, which meant that a decline in hardcore smoking was seen among smokers with high socioeconomic status but not among those with low socioeconomic status [5–7]. The absence of socioeconomic disparities in hardcore smoking in South Korea suggests that Korean tobacco control policies might have been effective across socioeconomic status.

### Implications

Hardcore smokers were more prevalent among males, middle age group, and never-married group but were not that different among socioeconomic groups. Although comprehensive tobacco control policies should cover the entire population, we should consider focusing on specific groups where hardcore smokers were more prevalent.

### Limitations

There are several limitations to this study. First, there is no consensus on the definition of hardcore smokers. We could use only three characteristics in the definition of hardcore smoking because other variables such as nicotine dependency, were not available from KNHANES. Second, there might be some social desirability bias in self-reported smoking behaviors. Because of the strong social disapproval for women who smoke in Korea [16], prevalence of female hardcore smoking might be under-reported. Third, we could not investigate smoking periods because surveys from 2007 to 2009 did not include duration of smoking. Lastly, given the absence of a decline in smoking prevalence during the study period, we could not examine the hardening hypothesis.

## Conclusion

We found that some subpopulations, including men, those in their middle ages, and those never-married, showed greater likelihood of being hardcore smokers. We need to strengthen the comprehensive tobacco control policy by targeting these vulnerable populations.

## Abbreviations

CI: Confidence interval
EU: European Union
KNHANES: Korea National Health and Nutrition Examination Survey
OR: Odds ratio
PSU: Primary sampling units
USA: United States of America
